# Voluntary Notification

**Published:** 1901-07-27

**Authors:** 


### Voluntary Notification.

So many authorities are now urging the desirability of including consumption among the notifiable diseases that some of the facts brought forward by Alderman McDougall, showing the uses to which an intelligent municipality can apply the information gained by notification, will be read with interest.

Early in 1899 a scheme for the voluntary notification of phthisis limited in the first instance to public institutions was submitted to the Sanitary Committee of the City of Manchester and was accepted by them. A highly qualified medical man, Dr. Coates, was appointed, in September 1899, to assist the Medical Officer of Health in administering the scheme. In March 1900 the City Council accepted the further proposal that medical practitioners should be invited to notify their cases, and a second medical assistant, Dr. G. F. McCleary, was appointed.

To assist the Medical Officer of Health in carrying out the scheme, besides the two assistant medical officers, there were available the services, in part, of 28 district sanitary inspectors, and of 19 health visitors belonging to the Manchester and Salford Ladies' Health Society, and to the Jewish Ladies' Health Society, voluntary organisations aided by the corporation. By these the cleansing and disinfecting work has been supervised, and a continuous supervision has also been exercised over cases once reported.

The number of cases notified from September 1899 to the end of March 1901 has been 2,338. Of these 1,710 have been notified from public institutions, 628 by private practitioners. During that period the number of deaths registered from phthisis has been 1,729. The total number of houses disinfected between March 31st, 1900, and March 30th, 1901, has been 2,306. Needless to say, many sanitary defects have been discovered and removed.

The methods of disinfection employed have been to disinfect articles of clothing by steam, while rooms have been disinfected by washing the floors, woodwork, etc., with soft soap and water, cleansing the walls down with dough (Esmarch's method), and limewashing the ceiling. If, however, the house is dirty, or the patient has not carried out careful personal precautions, or marks of sputum are visible on the floor or walls, the surfaces of the room are washed with a solution of chlorinated lime, 1½ oz. to the gallon. Milk of lime is used to the walls, instead of solution of chlorinated lime, when rooms occupied by consumptives are disinfected.

The probable source of infection has been determined in 731 cases out of 1,785 notified up to the end of December 1900, and these facts will in due course serve as the starting-points of fresh administration.

